# Enhancing Molecular Characterization of Dissolved Organic Matter by Integrative Direct Infusion and Liquid Chromatography Nontargeted Workflows

**DOI:** 10.1021/acs.est.4c00876

**Published:** 2024-07-03

**Authors:** Jessica Patrone, Maria Vila-Costa, Jordi Dachs, Stefano Papazian, Pablo Gago-Ferrero, Rubén Gil-Solsona

**Affiliations:** † Department of Environmental Chemistry, Institute of Environmental Assessment and Water Research (IDAEA), Spanish Council of Scientific Research (CSIC), Barcelona 08034, Spain; ‡ Department of Environmental Science (ACES, Exposure & Effects), Science for Life Laboratory, Stockholm University, Stockholm 106 91, Sweden; 3 National Facility for Exposomics, Metabolomics Platform, Science for Life Laboratory, Stockholm University, Solna 171 65, Sweden

**Keywords:** dissolved organic matter, LC-orbitrap, molecular fingerprinting, environmental water

## Abstract

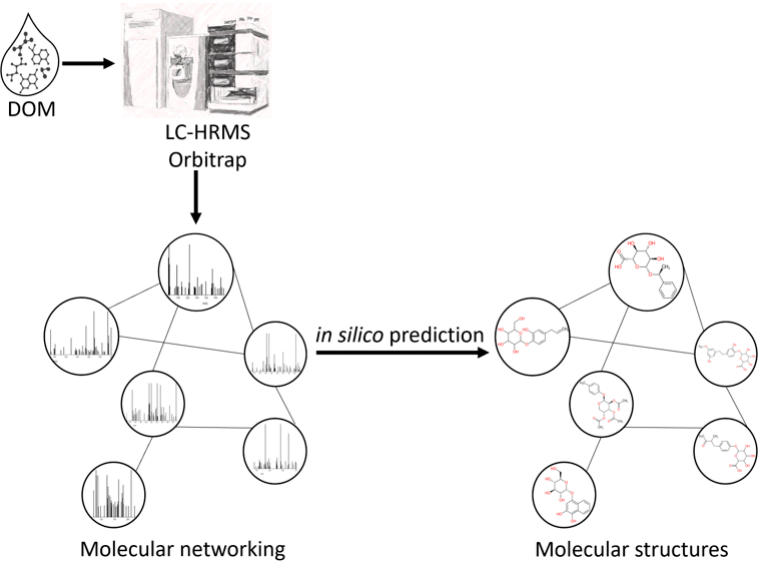

Dissolved organic matter (DOM) in aquatic systems is a highly heterogeneous mixture of water-soluble organic compounds, acting as a major carbon reservoir driving biogeochemical cycles. Understanding DOM molecular composition is thus of vital interest for the health assessment of aquatic ecosystems, yet its characterization poses challenges due to its complex and dynamic chemical profile. Here, we performed a comprehensive chemical analysis of DOM from highly urbanized river and seawater sources and compared it to drinking water. Extensive analyses by nontargeted direct infusion (DI) and liquid chromatography (LC) high-resolution mass spectrometry (HRMS) through Orbitrap were integrated with novel computational workflows to allow molecular- and structural-level characterization of DOM. Across all water samples, over 7000 molecular formulas were calculated using both methods (∼4200 in DI and ∼3600 in LC). While the DI approach was limited to molecular formula calculation, the downstream data processing of MS2 spectral information combining library matching and in silico predictions enabled a comprehensive structural-level characterization of 16% of the molecular space detected by LC-HRMS across all water samples. Both analytical methods proved complementary, covering a broad chemical space that includes more highly polar compounds with DI and more less polar ones with LC. The innovative integration of diverse analytical techniques and computational workflow introduces a robust and largely available framework in the field, providing a widely applicable approach that significantly contributes to understanding the complex molecular composition of DOM.

## Abstract 2

This work integrates diverse analytical techniques and computational workflows for a comprehensive understanding of dissolved organic matter, including structural-level characterization.

## Introduction

Dissolved organic matter (DOM) of aquatic systems is one of the largest reservoirs of organic matter on a global scale. DOM is operatively defined as the fraction of aqueous organic matter not retained on a 0.7 μm pore size filter and is constituted by a large, complex, and highly heterogeneous mixture of water-soluble organic compounds which are mainly biogenic^1^ but also include a minor fraction of anthropogenic origin.^2^ Concentrations of DOM generally range from 40 μM (<1 mg/L) in the deep ocean^3^ up to several hundred μM in impacted continental waters.^4^ DOM composition influences the function and activity of microorganisms involved in biogeochemical cycles and planetary ecosystem processes.^3,5,6^ A comprehensive chemical evaluation of DOM is particularly challenging due to the heterogeneity of its composition. Understanding DOM composition is crucial for assessing Earth ecosystem’s health, which should ideally encompass unraveling the anthropogenic impact on this organic matter pool, an issue often overlooked when characterizing DOM, despite recent advancements in nontargeted approaches for widescope screening of anthropogenic chemicals.

The development of high-resolution mass spectrometry (HRMS) approaches in combination with advanced computational tools has been increasingly and successfully applied to characterize DOM resolving a broad range of chemicals.^7−10^ Fourier transform ion cyclotron resonance mass spectrometry (FT-ICR-MS) has been the gold standard in DOM characterization, mainly thanks to its extremely high resolution of 1 000 000 full width at half-maxima (fwhm), enabling to resolve highly complex mixtures in DOM by allowing identification of thousands of molecular formulas.^11−16^ While increasingly adopted for DOM characterization, FT-ICR instruments have low availability and high operational costs, restricting their access to a limited number of research groups, which in turn limits the impact of HRMS in the DOM characterization field and the environmental sciences. More recently, benchtop Orbitrap mass spectrometers that can operate at nominal resolutions nearer FT-ICR, i.e., more than 450 000 fwhm (at *m*/*z* 200), have reached a wider user availability for a range of applications requiring high resolving power in environmental chemistry and biogeochemical investigation. The high-resolution Orbitrap MS has been applied before for DOM characterization, and compared with FT-ICR-MS,^17^ and thus been employed for DOM characterization in various matrices, including different aquatic systems.^18−21^

DOM characterization by HRMS is commonly performed by direct infusion (DI) full-scan acquisition employing electrospray ionization (ESI) operating in negative mode because of the high organic acids content of DOM.^22−24^ Although consolidated DI-HRMS methods for DOM analysis provide important knowledge of the elemental composition for a significant organic fraction of DOM, its application typically suffers from two major limitations: the signal suppression of multiple interfering analytes and overlapping mass spectra for isomers and isobars, and the lack of acquisition method and downstream data analysis that would allow for resolving the fine structural information on the complex chemical composition.

As an alternative and complementary approach to DI, liquid chromatography (LC) separation can provide new insights for a complementary characterization of DOM by introducing a new dimension to the analysis, although a significant obstacle is still represented by the high isomeric complexity of the DOM.^25,26^ Indeed, despite the ability to calculate thousands of molecular formulas, the elucidation of structural composition remains a major challenge in DOM characterization. The use of high-resolution MS2 contributed by collision-induced dissociation (CID) and high-energy collisional dissociation (HCD), allows to clarify certain structural aspects by the scrutiny of fragmentation patterns, and description of the functional groups of the assigned formulas via neutral losses depending on the MS techniques,^8,16,27−31^ including ion mobility mass spectrometry (IMS)^32,33^ and LC-Orbitrap.^34,35^ Different MS2 acquisition strategies are available for obtaining fragmentation spectra in a nontargeted workflow when using LC-HRMS. Data-dependent acquisition (DDA) is known to provide the highest quality of MS2 spectra^36^ and was used in LC-HRMS for nontargeted mapping of seawater DOM,^37,38^ in combination with small-molecule annotations by MS2 spectral library matching and molecular networking.^39,40^ However, DDA is biased toward higher abundant species and excludes less abundant ions based on precursor ion intensity, making it not ideal for the trace-level analysis of anthropogenic organic contaminants in the environment, or biogenic chemicals with low abundances. In comparison, data-independent acquisition (DIA) provides a more unbiased representation of analytes by acquiring MS2 spectra for all ions within a specific mass range and is thus arguably the method of choice for nontargeted molecular characterizations of complex environmental matrices. In a recent study, the effectiveness of this approach was successfully demonstrated via the integration of DIA nontargeted HRMS (Orbitrap) analysis with molecular networking and high-throughput in silico structure annotation,^41^ which together provided molecular-level insight into the characterization of complex airborne pollution on fine particulate matter.^42^

Despite instrumental developments, new computational approaches are now available that further enhance structural understanding of DOM when, for instance, integrated with traditional approaches.^43^ These allow for performing high-throughput functional group characterization, combining large-scale computational calculation of neutral losses using the mass fingerprint difference between precursors and fragments. However, these approaches have remained limited to a narrow set of targets. Indeed, these approaches lack a widescope approach that includes a comprehensive structural elucidation workflow. Here, we aim to take advance of the cutting-edge and widely accessible hardware, as benchtop high-field Orbitrap with faster scanning acquisition (DIA), as well as data science tools in chemometrics (i.e., in silico structural elucidations) to gain a deep structural-level characterization of DOM.

The overall objective of this study was thus to assess the potentiality of a nontargeted workflow that enables the integration of DI-HRMS with LC-HRMS (including ESI in both positive and negative modes to broaden the spectrum of analysis) combined with downstream novel data treatment and computational tools for the molecular and structural characterization of DOM. We demonstrate the applicability of this workflow for different representative waters with increasing degrees of chemical complexity, such as commercial bottled drinking water, coastal seawater, and river water from a highly urbanized Mediterranean area.

## Materials and Methods

### Sample Collection

The proposed protocol was applied to samples of coastal seawater, river water, and bottled drinking water. River water was sampled from the Llobregat River (Barcelona, 41°20′52.7″N 2°03′11.5″E), a Mediterranean fluvial system receiving significant anthropogenic pressure from the Barcelona metropolitan area. Seawater was sampled on the Castelldefels coast, south of Barcelona (41°15′50.4″N, 1°58′04.1′′E). Riverine and coastal samples were both collected at 0.5 m depth in glass bottles. Samples were immediately refrigerated and transported to the laboratory for processing. All the glass material used for sample collection and processing had been previously baked at 450 °C for 4 h. Drinking water was obtained from a local brand of mineral bottled water. Suwannee River fulvic acid (SRFA), obtained by the International Humic Substance Society (IHSS), was used as DOM reference material to ensure validation and reproducibility of the methods. Standard preparation and detailed results are described in the Supporting Information.

### Sample Extraction

**Figure 1:**
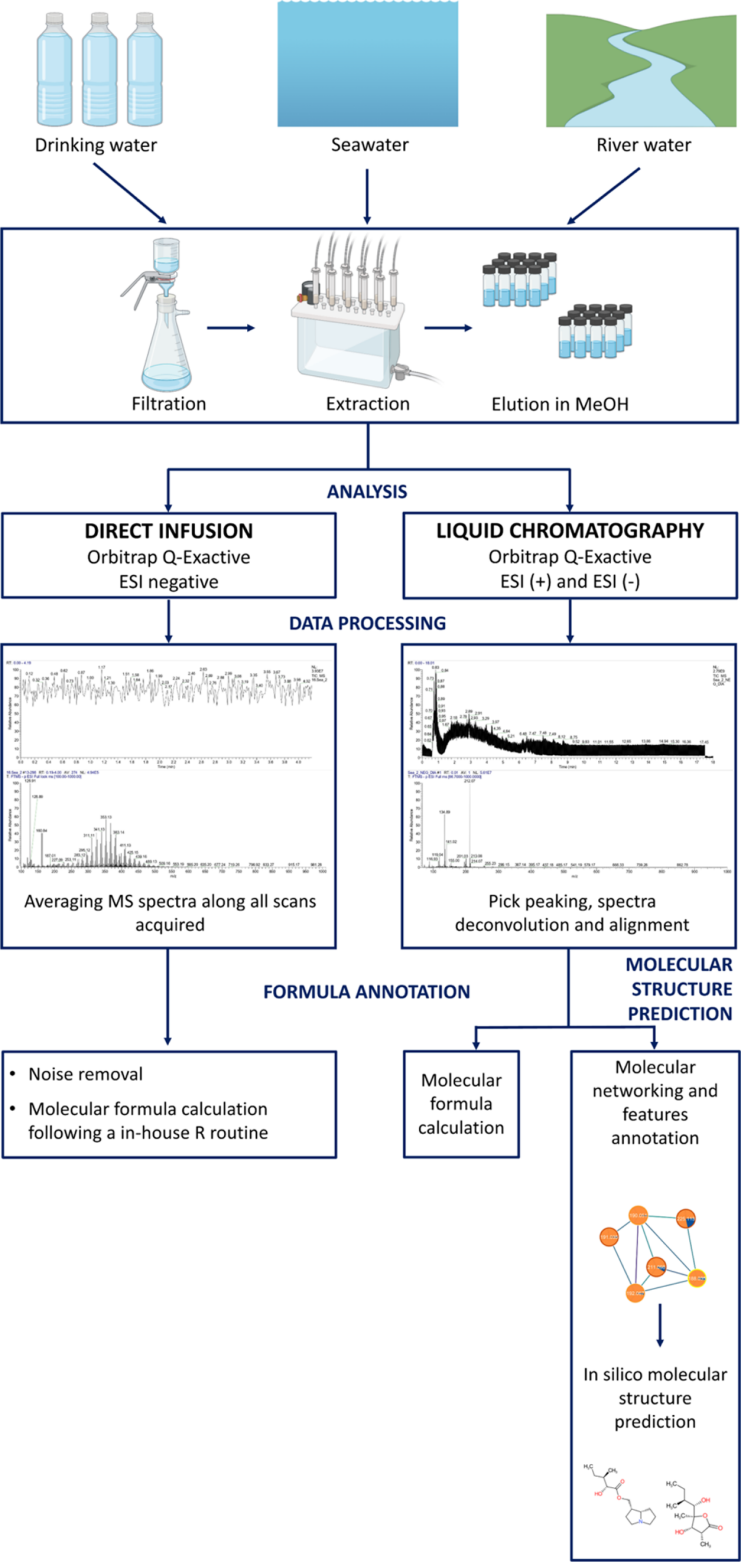
Experimental workflow. Samples from three different sources, i.e., seawater, river water, and drinking water, were filtrated, extracted, and processed by two HRMS acquisition methods (DI and LC-DIA). Raw data analysis included preprocessing, molecular formula calculations, and, for LC data, computational workflows of molecular networking and in silico structure prediction.

Details about the chemicals and extensive descriptions of the sample treatment protocols are provided in the Supporting Information. All of the samples were filtered and extracted in triplicates following a slightly modified protocol described by Dittmar et al. Briefly, river water (500 mL), seawater (2000 mL), and drinking water (2000 mL) were filtered through 0.7 μm Whatman GF/F glass microfiber filters, acidified to pH 2.0 by adding hydrochloric acid and extracted with PPL cartridges (500 mg, 3 mL; Agilent Technologies). Elution was performed with 3 mL of methanol (MeOH). Previous studies have shown that the SPE-DOM extraction efficiency when using PPL cartridges is about 60% of the total fraction.^22,44−47^ From each sample, two aliquots of 1 mL were prepared, one for DI analysis and one for LC analysis. Along these main samples, method blank samples (*n* = 3) were processed following the same protocols to monitor potential contaminations. Following solid-phase extraction, samples were analyzed by DI and LC coupled to HRMS, and data were processed following the flow summarized in Figure 1.

### DI-HRMS Instrumental Analysis and Data Preprocessing

For DI-HRMS analysis, samples were directly infused into a Q-Exactive Orbitrap (Thermo Fisher Scientific, Dreieich, Germany) employing electrospray ionization (ESI) in negative mode and data acquired in MS1 full scan (*m*/*z* range 100–1000 Da, at 70 000 nominal resolution fwhm at 200 *m*/*z*). The ESI source and the instrument ion optics setting were as follows: spray voltage −3.10 kV; sheath gas flow rate, 28; capillary temperature, 275 °C; syringe pump flow rate, 8 μL/min. Three hundred spectra were acquired for each sample. Samples were loaded in the ESI by Hamilton syringe (Thermo Scientific) using a syringe pusher. The syringe’s inner diameter was set at 2.3 mm. A whole needle volume of MeOH was injected after the acquisition of each sample to clean the source. The syringe and capillary were cleaned 10 and 2 times with grade MeOH to avoid contamination.

The acquired mass spectra from DI-HRMS were obtained by averaging 100 spectra along 3 min of acquisition using the software Thermo Xcalibur. A mass range between 200 and 800 Da was considered. Each sample feature’s mass list was individually processed to remove noise peaks below the detection limit (calculated considering the first 95th percentile of peaks with a mass defect ranging from 0.4 to 0.8 Da). The intensity of the peaks was then linearly correlated with their corresponding *m*/*z* values and was then processed in *R* for the molecular formula calculation.^48^ Signal intensities were normalized by summing all of the signals after removing the peaks detected in the blank. Only features present in at least two out of the three replicates of each water sample were taken into account.

### LC-HRMS: Instrumental Analysis and Data Preprocessing

For LC-HRMS analysis, chromatographic separation was conducted using a reversed-phase column (Cortecs C18+; 2.1 mm × 100 mm, 2.7 μm from Waters) included in a UHPLC system coupled to a Q-Exactive Orbitrap (Thermo Fisher Scientific) operated in both positive (ESI+) and negative (ESI−) mode in full-scan (*m*/*z* range 67–1000 Da, 70 000 nominal resolution fwhm at 200 *m*/*z*), with parallel all-ion-fragment data-independent (AIF/DIA) acquisition of MS2 spectra (*m*/*z* range 67–1000 Da, 70 000 resolution). More details about MS1 and MS2 acquisition are reported in Table S1. In positive ionization mode, the aqueous phase was water containing 0.1% formic acid and the organic one was MeOH containing 0.1% formic acid. In negative ionization mode, the aqueous phase consisted of water 5 mM ammonium acetate and the organic phase was MeOH 5 mM ammonium acetate. The flow rate was 0.3 mL/min, and the injection volume was set to 5 μL of sample. The Orbitrap system was equipped with an ESI, operating at 4000 V in positive and 3000 V in negative ionization modes, 350 °C capillary temperature, 40 sheath gas flow, 10 auxiliary gas flow, 100 of maximum spray current, 300 °C probe heater temperature, and 60 SLens RF level.

Data obtained from the injections were converted from proprietary (*.raw) to generic (*.mzML) format using ProteoWizard software,^49^ and then preprocessed into MS-DIAL (v.4.90)^50^ allowing molecular feature detection by MS1 chromatographic alignment across samples, peak integration, and DIA MS2 spectral deconvolution. Background contamination was excluded by using a sample-to-blank ratio threshold higher than 5 (maximum sample intensity/blanks average). Full details are reported in Table S2. Only features present in at least two out of the three replicates of each water sample were considered. The raw and processed MS data are publicly available at the MassIVE depository (https://massive.ucsd.edu/ProteoSAFe/static/massive.jsp); data set IDs: MSV000094540 and MSV000094541.

### Molecular Formulas Assignment for DI- and LC-HRMS Data

For DI-HRMS, molecular formulas were calculated using an in-house R routine. Formulas were allowed only if following these criteria: *m*/*z* between 200 and 800; maximum error 5.0 ppm; O/C ratios in the range 0–1; H/C ratios in the range 0.3–2.5; DBE-O (double bond equivalent minus O atoms) between −10 and 10; admitted atoms, C_4–40_H_1–80_O_1–40_N_0–1_ with and without one ^13^C. Notably, the R script used does not account for the presence of isotope patterns other than ^13^C. Other heteroatoms, namely, S and halogens, were also excluded in the formula calculation for DI-HRMS since they led to an unacceptable number of false assignments. Including S, for example, results in a composition of the samples with an unreasonable percentage of S-containing compounds, inconsistent with current estimates of organic S.^51^ Furthermore, it was not possible to confirm the presence of sulfur in the aforementioned compounds by manual inspection of the spectra.

For LC-HRMS, molecular formula assignments were performed in SIRIUS (v.5.6.2)^52^ which calculates fragmentation trees combining information from MS1 accurate mass, isotopic ratios, as well as MS2 spectral fragments. Tolerance for accurate mass was set to <5 ppm (SIRIUS default setting for Orbitrap HRMS). The elements allowed were C, H, O, N, S, Br, Cl, F, and P, considering only formulas in the available structural databases: Biodatabase, Biocyc, CHEBI, COCONUT, EcoCycMine, GNPS, HMDB, KEGG, KEGG Mine, KNApSAck, Natural Product, Plantcyc, PubChem, YMDB, YMDB Mine, ZINC bio.

### LC-HRMS: Molecular Networking and In Silico Structural Prediction

Molecular networking was built for clustering of features based on their respective MS2 spectral similarity using the Feature-Based Molecular Networking (FBMN) workflow^40^ within the Global Natural Products Social Molecular Networking (GNPS)^39^ platform. Networks parameters were set as follows: precursor ion mass tolerance and MS2 fragment ion tolerances of 0.01 and 0.05 Da, minimum spectra similarity cosines >0.65, and a minimum of 4 shared spectral peaks. The spectra in the network were then searched against GNPS public spectral libraries (https://gnps-library.ucsd.edu). For spectral library matching, annotations were considered with cosines >0.60 and a minimum of 2 shared spectral peaks. Molecular structures were predicted in GNPS using the in silico Network Annotation Propagation (NAP)^41^ based on the MetFrag algorithm^53^ and structural databases (GNPS, HMDB, SUPNAT, CHEBI, DRUGBANK, FooDB). In NAP, in silico candidates are reranked combining library matches, if present, and in silico MetFrag predictions. If library matches are few or absent, then structural similarity among candidate structures within the same network is prioritized and expressed as consensus scoring.^41^ The following parameters were used: 10 first candidates for consensus score, 5 ppm accuracy, cosine score >0.65, maximum of 10 candidate structures. The top-first candidate structure predicted by NAP has been taken into account. According to da Silva et al., the correct annotation rate within the first candidate is 30–50%. The assignment confidence in putative structures was increased by combining them with the calculated molecular formula. Molecular structures predicted by NAP were translated into formulas using OpenBabel 3.1.1^54^ and matched with molecular formulas previously calculated, only consensus formulas between SIRIUS and the first NAP prediction were taken into account. The molecular networks were visualized using Cytoscape software.^55^ The molecular networks and spectral library matching are available in GNPS (LC-ESI+: https://gnps.ucsd.edu/ProteoSAFe/status.jsp?task=8106ee70142748e1a4beb93d6cab4f25; LC-ESI–: https://gnps.ucsd.edu/ProteoSAFe/status.jsp?task=0e5b5773845f47dfb518b61e7809ddf3).

## Results and Discussion

### DOM Characterization Based on DI-HRMS Workflow

**Figure 2:**
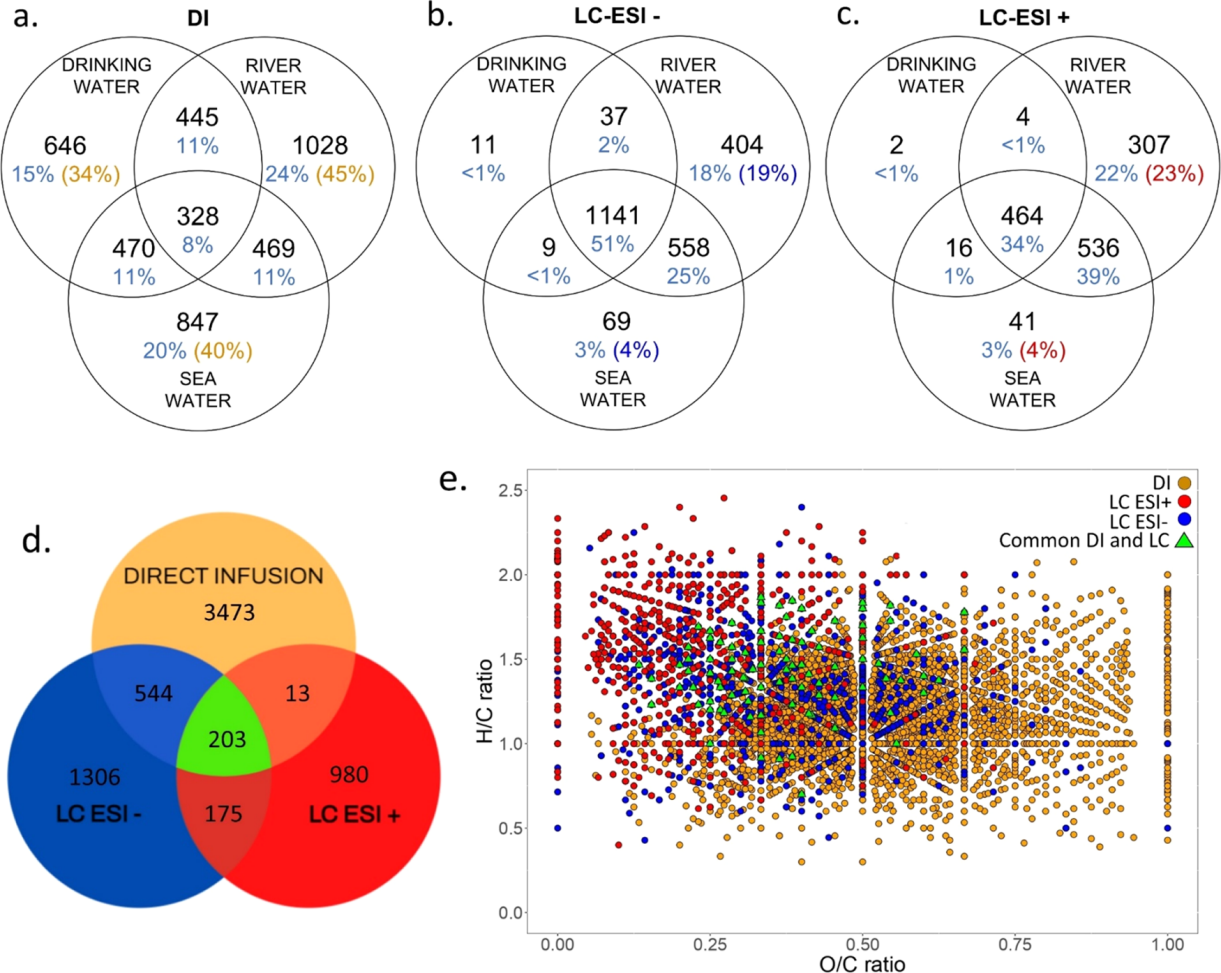
Molecular formulas distribution among samples and methods. Venn diagrams showing the formulas that were unique to each sample in direct infusion (a) and liquid chromatography in negative (b) and positive (c) ionization modes. The percentage of formulas considering all three samples is reported in blue, the percentage in the parentheses represents the % of formulas unique in that single sample type. (d) Venn diagram and (e) van Krevelen plot showing the overlap of the whole set of formulas assigned by DI and LC (DI in yellow, LC-ESI- in blue, and LC-ESI+ in red; green triangles represent the common compounds in the two methods DI and LC).

For each direct-injected sample, spectra comprised in the 200–800 Da range were extracted from the raw file. After noise subtraction, each sample replicate comprised an average of 4651 (±628), 4996 (±603), and 3847 (±1221) features for river, sea, and drinking water, respectively. For approximately 50% of these, a molecular formula was calculated, yielding 2270, 2114, and 1889 formulas for each water type, respectively. A total of 8% of these molecular formulas were common in the three matrices, suggesting a high degree of heterogeneity in the different DOM compositions (Figure 2a). Previous studies have shown a relevant variability in the molecular composition of features in seawater from different water masses.^56^ The larger variability observed here for freshwater, seawater, and drinking water is consistent with the fact that different water types were analyzed. The count of attributed formulas closely aligns with observations from prior studies employing DI-HRMS (Orbitrap) workflows, where the range of chemicals detected in seawater by molecular formula calculation spanned from 1500 to 3400.^21,23^ The distribution in the chemical space of molecular formulas annotated in DI can be visualized in the Van Krevelen diagram (Figure 2e). Each dot on the diagram represents one or more features based on its O/C and H/C ratios. The majority of formulas concentrate in the space between 0.3 < O/C < 0.6 and 1 < H/C < 1.5, mainly phenolic and lignin-like compounds (Figure S1).

### DOM Characterization Based on LC-HRMS Workflow

**Figure 3:**
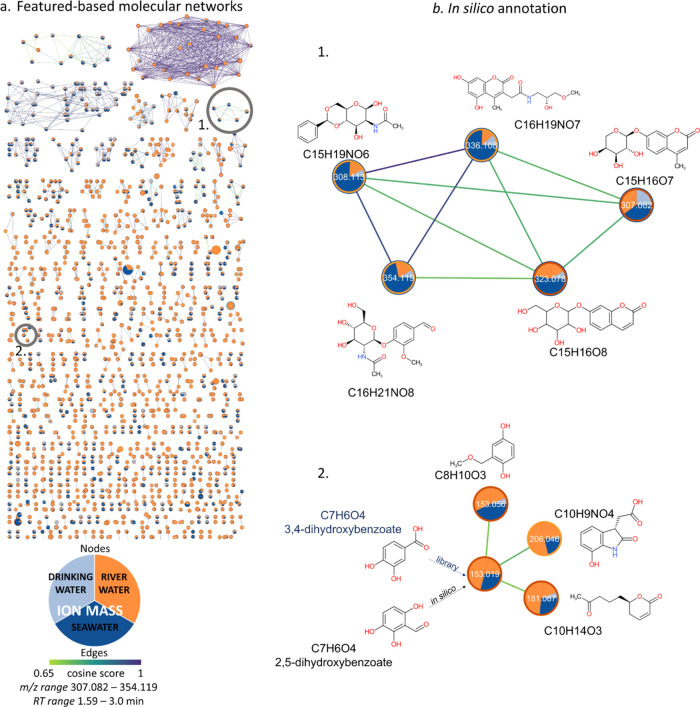
Molecular networks and in silico structural annotations. Molecular networks were built in GNPS based on MS2 spectra similarity. In this example, 17 796 features were clustered into 1789 molecular families (a). Zoom-in on a molecular family including five aromatic compounds (b). Each feature is represented as a node and colored by its relative abundance among the different samples, i.e., river water (orange), seawater (blue), and drinking water (light blue). Edges linking each node are colored based on the modified cosine score (>0.65) representing the spectral similarity between each feature (nodes). The molecular formulas and the associated first candidate structures from in silico predictions reranked by the MetFrag/NAP workflow are shown for some of the features in the example subnetwork clusters (b1, b2). The bottom cluster (b2) includes a node with putative annotation in the GNPS library (3,4-dihydroxybenzoate, *m*/*z* 163.019, RT 1.81 min). In this example, the first candidate structure from NAP is an isomer of the level 2 annotated compound (2,5-dihydroxybenzoate), while the 3,4-dihydroxybenzoate is predicted and ranked as third candidate.

A total of 16 033 molecular formulas (i.e., 10 132 in ESI– and 5901 in ESI+, Figure S2) were assigned, which corresponded to 72% of all features detected (after blank subtraction). Next, MS2 spectra information was leveraged to build molecular networks and predict molecular structures in silico within the GNPS ecosystem. In the FBMN networks, features in the networks are represented as nodes connected to each other based on their respective mass spectral (MS2) similarity, thus inferring a structural analogy (Figure 3). Less than 1% of features could be annotated via MS2 spectral library matching on the GNPS large public spectral database (all library matches are accessible on the GNPS platform, see links to FBMN jobs reported in the Materials and Methods section). By simultaneously exploiting spectral library annotations and the neighbor connections in the molecular networks, NAP allowed extending structural annotations in silico to the remaining portion of the data set. In total, the structures of 13 584 (9029 in ESI– and 4555 in ESI+) molecules were predicted among all of the water samples. A conservative approach was applied by further considering only those in silico structural candidates suggested by NAP that were consistent with the previously assigned formula by SIRIUS. Therefore, 3599 (2228 in ESI– and 1371 in ESI+) molecular formulas were finally supported by a structure, representing 16% of the entire LC-HRMS data set. These results are consistent with a previous application of the same workflow developed for the nontargeted molecular characterization of airborne PM_2.5_.^42^ Comparison of results from both software (SIRIUS and NAP) reduced the number of potential identifications, but it increased reliability and consistency with the expected composition of the samples (details in the following section).

The table summarizing the results, including any assignments in the library, is reported in Supporting Data 1. The molecular formulas of compounds annotated on the MS2 spectral library (24) were found consistent with the molecular formula of the NAP first candidate for 79% (19/24) of the cases, while the first candidate structure corresponded to the correct compound or a similar positional isomer (e.g., 3,4-dihydroxybenzoate and 2,5-dihydroxybenzoate in Figure 3b) for 42% (8/19) of the in silico annotations.

**Figure 4:**
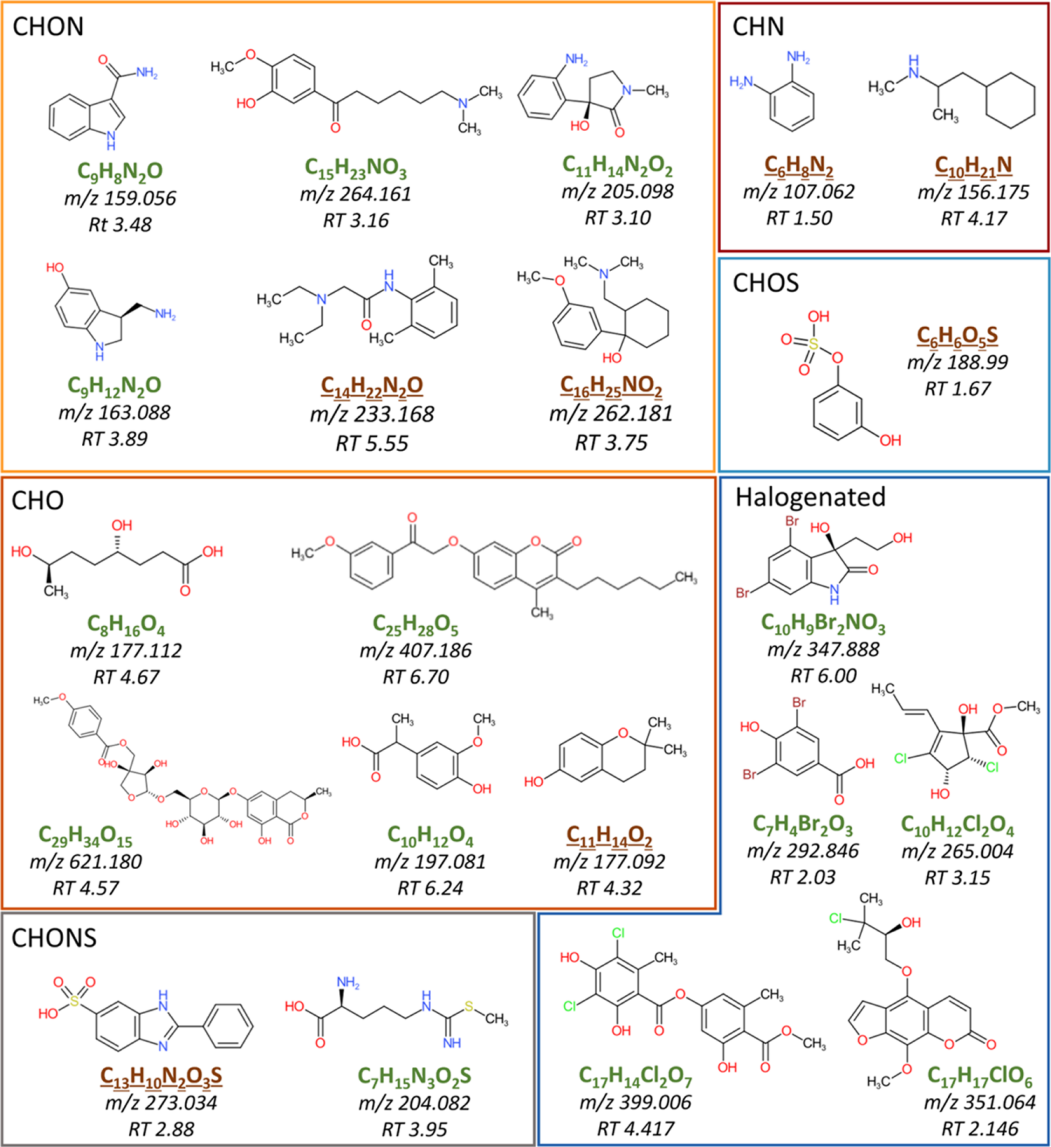
Examples of in silico predicted candidate structures. Molecular formulas in green represent natural compounds or metabolites, while formulas in brown and underlined can be attributed to environmental contaminants, according to the information provided for each structure by NAP databases.

Despite the lack of confirmation or consensus with library matches for candidate structures, resulting in Level 3 annotation, this analytical and downstream approach yields valuable insights into the chemical and structural attributes of the examined mixture. The same LC workflow was employed with DDA acquisition (details provided in the Supporting Information). As expected, the overall number of library matches was higher than in DIA, achieving approximately a 17% Level 2 annotation (123 annotations out of 740 total molecules in LC-ESI+ and ESI−). However, the total amount of detected compounds and potential structural candidates was notably lower (740 out of 3600). This underscores the superior quality of MS2 spectra in DDA and consequently a heightened library match rate. Nevertheless, the method demonstrated limitations in achieving comprehensive structural characterization. Hence, while greater confidence is placed in library matches, LC-DDA contributed less to the characterization of DOM.

### Complementarity of DI and LC-HRMS Approaches

**Figure 5:**
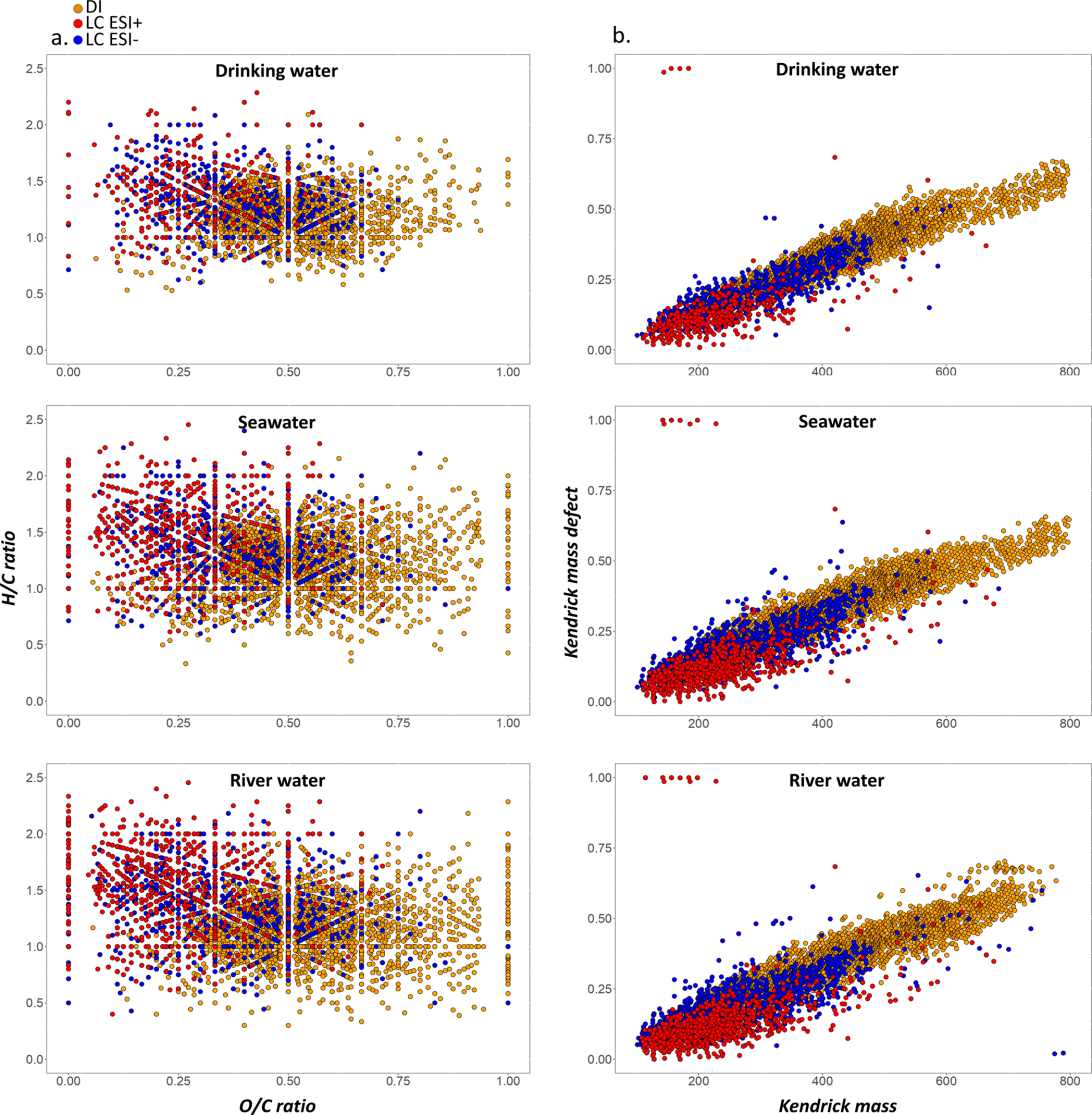
(a) Van Krevelen diagrams showing assigned peaks in seawater, drinking water, and river water samples by DI-HRMS (orange), LC-(ESI−)HRMS (blue), and LC-(ESI+)-HRMS (red). (b) Corresponding Kendrick diagrams of data described in van Krevelen areas of (a).

With LC (ESI+ and ESI−) analysis, 13 888, 11 592, and 5357 molecular formulas were calculated in river, sea, and drinking water, respectively. Out of these, the structures of 3462 (25%), 2822 (24%), and 1683 (31%) compounds were predicted. These last are displayed in van Krevelen diagrams alongside the molecular formulas assigned by DI according to each water sample (Figure 5a) and the whole data set (Figure 2e). The minor overlap (3%) between chemical classes for compounds detected by DI and LC supports that different DOM fractions are covered by each method (Figure 2d). The LC (ESI+ and ESI−) was more selective for compounds with a low O/C ratio and high H/C ratio (Figures 2e and 5a), meaning fewer polar chemicals, while the DI mainly covered the chemical space included between O/C > 0.5 and 0.5 < H/C < 1.5, indicating a higher oxygen content and therefore higher polarity of compounds in DI than by reversed-phase LC. As also observed by Patriarca et al., this is most likely due to the lower ion competition during the ESI after the chromatography which leads to less ionizable species being detected more efficiently than in DI.

The chemical profiles generated by the two approaches are also shown in the Kendrick mass plots (Figure 5b), where compounds are distributed according to their masses and homologous series of chemically related compounds are distributed horizontally. In the DI analysis, *m*/*z* values range from 200 to 800 Da, while in LC analysis, the mass range was extended from 100 to 1000 Da. It was expected that LC would identify a higher number of low-molecular-weight compounds. However, despite a moderate fraction of compounds with >500 Da (20% of the total detected), the annotation at the structural level of those high-molecular-weight compounds is relatively limited (only 2%, while having the molecular formula of 60% of them). This means that a significant portion of large molecules is excluded when considering only the features that are consistent between SIRIUS and NAP and so the assignment of molecular formulas for high-molecular-weight compounds differs between the two methods.

Based on these results, DI and LC provide different and complementary information about organic matter composition. DI detects and assigns more molecular formulas, covering a wide chemical space and including high-molecular-weight and most polar compounds, but the information is limited to elemental composition, and only a few elements can be considered. Differently, LC is focused on the detection of less polar species when using typical C18 chromatographic columns, especially when including positive ionization, and for these compounds, it allows molecular formula assignation and candidate molecular structure prediction.

### Molecular Profiles of DOM across Different Water Sources

In order to define and test the applicability of the workflow on different DOM pools, water samples were examined, from three different sources corresponding to different degrees of DOM content, i.e., comparing drinking water and seawater (<1 mg/L DOM) with several mg/L river water (>1 mg/L DOM). The different chemical composition between the samples was evident in terms of the total number of detected features and the chemical classes. The 51% (ESI−) and 34% (ESI+) of the compounds detected by LC were common among the three sample types (Figure 2b,c), differently in DI, the 8% of compounds are shared (Figure 2a).

The detected features were classified according to their elemental composition as CHO, CHON, CHONS, and CHOS, including additional heteroatoms such as halogens or sulfur (in the case of the LC approach) (Table 1). Water samples from the Llobregat river, which is a water body that receives a high number of relevant anthropogenic inputs (i.e., wastewater effluents), had the largest number of compounds detected and showed a higher intensity of heteroatomic formulas. Among these, by cross-referencing the data of compounds for which an in silico structure has been predicted with those of compounds commonly detected in urbanized rivers according to Wilkinson et al.,^57^ pharmaceutical products such as lidocaine (C_14_H_22_N_2_O), tramadol (C_16_H_25_NO_2_), and pregabalin (C_8_H_17_NO_2_) were putatively annotated.

**Table 1:** Elemental Composition of the Samples in Terms of Number and Relative Intensity of Features for Which a Molecular Formula Was Calculated According to the Different Analytical Methods (X Indicates Halogens, nd Stands for Not Detected, “-” Stands for Formulas Not Considered)

	method	DI-HRMS			LC-ESI(−)-HRMS			LC-ESI(+)-HRMS		
	sample	seawater	drinking water	river water	seawater	drinking water	river water	seawater	drinking water	river water
% features	CHO	64.95	71.68	65.77	72.82	83.54	67.51	44.02	50.62	41.04
	CHO-X	-	-	-	0.96	0.33	1.17	0.19	nd	0.15
	CHON	35.05	28.32	34.23	17.84	11.95	20.94	49.28	45.68	48.07
	CHON-X	-	-	-	0.62	0.42	0.89	0.57	0.62	0.68
	CHOS	-	-	-	4.22	2.51	4.11	0.48	nd	0.53
	CHOS-X	-	-	-	0.06	nd	0.05	nd	nd	nd
	CHONS	-	-	-	2.64	1.00	3.93	1.15	1.03	2.27
	CHONS-X	-	-	-	0.23	0.08	0.33	nd	nd	0.30
	CHN	-	-	-	0.39	0.08	0.84	4.31	2.06	6.95
	CHN-X	-	-	-	0.06	0.08	0.14	nd	nd	nd
	P-cont	-	-	-	0.17	nd	0.09	nd	nd	nd
% intensity	CHO	68.87	84.82	55.73	82.52	98.27	66.15	66.10	66.79	17.97
	CHO-X	-	-	-	0.18	0.04	1.15	0.05	nd	0.00
	CHON	31.13	15.18	44.27	8.18	1.20	17.57	31.54	32.10	77.03
	CHON-X	-	-	-	0.02	0.05	1.05	0.03	0.07	0.14
	CHOS	-	-	-	1.57	0.15	9.30	0.11	nd	0.36
	CHOS-X	-	-	-	0.00	nd	0.01	nd	nd	nd
	CHONS	-	-	-	7.32	0.23	3.85	0.17	0.03	0.43
	CHONS-X	-	-	-	0.07	0.00	0.08	nd	nd	0.08
	CHN	-	-	-	0.04	0.00	0.79	2.00	1.01	3.99
	CHN-X	-	-	-	0.07	0.06	0.03	nd	nd	nd
	P-cont	-	-	-	0.03	nd	0.02	nd	nd	nd

The LC was more efficient in detecting heteroatomic compounds, particularly those containing S and halogens, which were instead excluded from the DI analysis. This is most likely due to the better ionization in the absence of charge competition in the source, which occurs after chromatographic separation. These compounds are mainly those containing one or more nitrogen atoms, particularly aromatic and N-heterocyclic compounds of both natural and anthropogenic origin. More N-containing formulas have been found in river water compared to seawater and drinking water. This is consistent with observations from other studies^58^ where a decrease of CHON formulas was observed along a river-to-sea transect and associated with the increasing salinity. According to Yan et al., a gradual increase in the degree of saturation and aromaticity was also observed with the increasing number of nitrogen atoms per formula.

S-containing compounds were considered exclusively with the LC method and were mainly found in river water, and to a lesser degree in seawater. Some examples are represented by compounds putatively annotated through their structure as the sunscreen agent Ensulizole (C_13_H_10_N_2_O_3_S), found at high intensity in seawater samples and in river water, and 2-benzothiazolesulfonic acid (C_7_H_5_NO_3_S_2_), found in river water. The presence of these compounds was further confirmed with standards as shown in Figure S4. Examples of some in silico predicted structures are shown in Figures 3 and 4.

### Perspectives and Applications

DOM is a highly complex mixture of natural and anthropogenic compounds and its comprehensive analysis requires the integration of multiple analytical techniques. Here, we demonstrate the application of comprehensive workflows to enhance the characterization of DOM in water samples by combining dedicated solvent extraction, DI- and LC-HMRS instrumental analysis, and novel computational data analysis to achieve an in-depth structural determination of DOM chemical components. This represents a significant advancement and innovation in DOM studies and paves the way for a more comprehensive characterization of chemical profiles. Analysis by DI-HRMS allowed the detection of a higher number of compounds covering a wide chemical space, especially toward more polar species, but provided limited information compared to data acquired by LC-HRMS (DIA) which can be integrated more efficiently with downstream computational workflows. Although reversed-phase LC-HRMS has a narrower and more selective chemical coverage compared to DI, it allows generation of information-rich data that can be leveraged for high-throughput structural characterization. The novelty in LC-HRMS use arises from the application of in silico characterization in the analysis of complex mixtures in aquatic environments. This represents an advancement as it broadens the scope of characterization, utilizing tools and instrumentation that are widely accessible. Despite the MS2 spectral library matching allowing only 1% annotations (Level 2), the computational tools applied here allowed extending annotations to in silico structural characterization (Level 3) up to 16% of the LC-HRMS data set. The potential to increase Level 2 annotations was observed by employing additional LC-DDA acquisition. However, this approach significantly reduced the number of structural candidates, which are valuable information for expanding our understanding of DOM composition. Indeed, the structural characterization potentially enables the prediction of physicochemical properties and reactivity of the species detected. With a more specific characterization of differences in the DOM mixture, it would have the potential, for example, to identify and classify the type of organic matter, investigate the natural or anthropogenic origin of its formulation, evaluate the changes in its composition, and correlate these characteristics with the biogeochemical cycle trend and with the presence of specific bacterial communities.

The use of a sole LC-HRMS method is still not exhaustive to disentangle the DOM complexity since it is still limited to a relatively small part of the organic fraction. In this sense, DI and LC arise as complementary approaches. Future research efforts should investigate the use of different stationary phases (e.g., HILIC) to extend the chemical space when using LC. The integration of an LC-Orbitrap approach with the presented workflows would further enhance the characterization of organic matter if used complementarily with DI-Orbitrap but it could also implement FT-ICR-MS approaches, given that the covered chemical space tends to encompass only the most polar fraction of compounds in this instance as well.

## Supporting Information Available

Detailed results of the features detected in the samples by LC-HRMS (DIA), with the respective library annotations and putative candidate structures (Supporting data2) (XLSX)
Detailed results of the features detected in the SRFA reference material by DI-HRMS and LC-HRMS (DIA) (XLSX)
Chemicals and materials; additional experimental details and parameters (Tables S1 and S2); reference material analysis; additional plots on sample composition; and MS spectra of compounds confirmed with standards (Supporting data1) (PDF)
