# Supplementary material for: Enhancing Molecular Characterization of Dissolved Organic Matter by Integrative Direct Infusion and Liquid Chromatography Nontargeted Workflows
Source: Environ Sci Technol. 2024 Jul 3;58(28):12454–66. doi: 10.1021/acs.est.4c00876 (PMC11256763; doi:10.1021/acs.est.4c00876)
Supplement: Supplementary file 3 — es4c00876_si_003.pdf [file es4c00876_si_003.pdf]

SUPPORTING INFORMATION TO:

Enhancing Molecular Characterization of Dissolved Organic Matter by Integrative Direct Infusion and Liquid Chromatography Non-Targeted Workflows

Jessica Patrone[a], Maria Vila-Costa[a], Jordi Dachs[a], Stefano Papazian[b], Pablo Gago-Ferrero[a], Rubén Gil-Solsona*[a]*

[a] Department of Environmental Chemistry, Institute of Environmental Assessment and Water Research (IDAEA), Spanish Council of Scientific Research (CSIC), Barcelona 08034, Spain

[b] Department of Environmental Science (ACES, Exposure & Effects), Science for Life Laboratory, Stockholm University, Stockholm, 106 91, Sweden

*Email: ruben.gil.solsona@csic.es
*Email: pablo.gago@idaea.csic.es

CONTENT:

SI-1: Chemicals and materials

SI-2: Sample treatment

SI-3: SRFA analysis by DI-HRMS and LC-HRMS

SI-4: LC-HRMS analisys in DDA acquisition mode

Table S1: Q-Exactive Orbitrap MS parameters for LC-HRMS analysis

Table S2: MSDIAL parameters

Figure S1.

Figure S2.

Figure S3.

Figure S4.

SI-1: Chemicals and materials

Water and methanol (LiChrosolv® LC/MS grade) for solid phase extraction were acquired from Merck (Darmstadt, DE). Water and methanol (Optima® LC/MS grade) for instrumental analysis were purchased from Thermo Fisher Scientific. Hydrochloric acid 25% (ACS grade) was purchased from Merck (Darmstadt, DE). Glass fiber filters (GF/F, 0.7 µm mesh size, 47 mm diameter) were purchased from Whatman (Little Chalfont, UK). SPE cartridges with a styrene-divinylbenzene stationary face Bond Elut PPL (Priority Pollutant) were acquired from Agilent Technologies (Santa Clara, USA). The reference material Suwanne River Fulvic Acid (SRFA, 2S101F) was purchased from the International Humic Substances Society.

SI-2: Sample treatment

Samples were filtered through 0.7-µm Whatman GF/F glass fiber filters immediately after the collection, acidified to pH 2.0 by adding hydrochloric acid stored at -20°C overnight. Before the extraction, PPL cartridges were washed three times with 1 mL of methanol, soaked overnight with 3 mL of methanol and conditioned with 3 mL of HPLC grade water acidified to pH 2 by adding hydrochloric acid. Samples were loaded under vacuum at about 3 mL/min. Cartridges were then washed with 3 ml of acidified HPLC-grade water and dried. Elution was performed with 3 ml of methanol and the final extract was collected in chromatographic vials, previously weighed. Two aliquots of 1 mL of each sample were prepared, one for direct infusion analysis and one for liquid chromatography. Samples were stored at -20°C until the day of the analysis. On this day each sample was diluted 1:1 with ultrapure water. The SRFA powder was weighed and diluted to 50 mg/L in MeOH:HPLC grade water (1:1).

SI-3: SRFA analysis by DI-HRMS and LC-HRMS

The reference material SRFA was analyzed by DI-HRMS and LC-HRMS following the same conditions as the other samples. A procedural blank was injected at the same time and the blank signals were removed during the data processing. Following the recommendations of the interlaboratory comparison study on DOM composition (Hawkes et al, 2020), results were compared through the online data comparison tool here provided (https://kairos.warwick.ac.uk/InterLabStudy) to facilitate the comparison of DOM reference standards. Raw data are available in the MassIVE depository (MSV000094643) and results are reported in **Supplementary_data2**.

For the molecular formula calculation of SRFA sample injected in DI, the following rules were respected: maximum error 5.0 ppm; O/C ratios in the range 0–1; H/C ratios in the range 0.3–2.5; DBE-O (double bond equivalent minus O atoms) between −10 and 10; m/z between 200 and 800; admitted atoms, $C_{4-40}H_{1-80}O_{1-40}\,N_{0-1}S_{0-1}$ with and without one ^{13}C.
Peaks with assigned molecular formulas detected amounted to 3453. Of these, 1071 were found to be in common with the reference dataset provided in InterLabStudy, yielding a 95% match. The corresponding metric data were calculated as follows: H/C metric, 1.1008; O/C metric, 0.5344; AImod metric, 0.3256, and MW metric, 403.4267. These results indicate a consistency of our DI-HRMS method with previous approaches used to characterize DOM.

The SRFA sample injected by LC-HRMS was processed according to the workflow described in the main document. The novelty of its application makes these results not strictly comparable with previous studies. A molecular formula and a structure candidate were attributed to 573 features. In this case, only 2% of common peaks were observed in the comparison with InterLabStudy. **Figure S3** shows the VK diagram of these results confirming what was observed

in the samples of the present study as well. The LC analysis and workflow here developed, only focus on a fraction of the organic matter that differs from the one detected by DI-HRMS methods and with which has a very little overlap.

SI-4: LC-HRMS analisys in DDA acquisition mode

The chromatographic separation was performed in the same conditions as the DIA experiment. The UHPLC system was coupled to Q-Exactive Orbitrap (Thermo Fisher Scientific) operated in both positive (ESI+) and negative (ESI-) mode in full-scan (m/z range 67-1000 Da, 70000 nominal resolution FWHM at 200 m/z), with parallel data-dependent (DDA) acquisition of MS2 spectra from the top 5 most abundant ions per cycle (m/z range 200-1000 Da, 35000 resolution, normalized collision energy 35). Full details are reported in **Table S1**. Data were processed as in the DIA experiment, including formula calculation, featured-based molecular networking, and network annotation propagation. Raw and processed data are available in MassIVE (MSV000094642). Molecular networks and library matches are available in GNP (LC-ESI+:link; LC-ESI-:link).

A total of 740 features were associated with a formula and a respective molecular structure (714 in river water, 641 in seawater, and 341 in drinking water). Of these, 123 (17%) were annotated in the GNPS libraries. With this approach, more library annotations are gained, but the overall number of compounds is five times lower than in DIA, resulting in a loss of structural information and very low coverage of the DOM chemical space.

Table S1: Q-Exactive Orbitrap MS parameters for LC-HRMS analysis

**Full MS / DIA MS2**		**Full MS / DDA MS2**	
Full MS	Runtime: 0 to 18 min<br>Polarity: positive and negative<br>Microscans: 1<br>Resolution 70000<br>AGC target: 3e6<br>Maximum IT: 150ms<br>Scan range: 66.7 to 1000 m/z	Full MS	Runtime: 0 to 18 min<br>Polarity: positive and negative<br>Microscans: 1<br>Resolution 70000<br>AGC target: 1e6<br>Maximum IT: 150ms<br>Scan range: 66.7 to 1000 m/z
DIA MS2	Microscans: 1<br>Resolution 70000<br>AGC target 3e6<br>Maximum IT: 150ms<br>(N)CE: 10; 40<br>Scan range: 66.7 to 1000 m/z	DDA MS2	Microscans: 1<br>Resolution 35000<br>AGC target 1e5<br>Maximum IT: 60ms<br>TopN: 5<br>Isolation window: 2.0 m/z<br>Scan range: 200 to 1000 m/z<br>(N)CE: 35<br>Minumum AGC target: 1e2<br>Intensity threshold: 1.7e3<br>Apex trigger: 3 to 10s

Table S2: MSDIAL parameters

MSDIAL PARAMETERS	
Data type	Centroid data
Data Collection	MS1 tolerance: 0.001 Da<br>MS2 tolerance: 0.005 Da<br>MS1 mass range: 90 -1000 Da<br>MS/MS mass range: 90-1000<br>Maximum charge number: 2
Peak detection	Minimum peak height 100000 amplitude<br>Mass slice width 0.05 Da<br>Smoothing method: Linear weighted moving average<br>Smoothing level 3 scan<br>Minimum peak width 10 scan
MS2Dec	Sigma window value 1<br>MS/MS abundance cut off 10 amplitude<br>Keep isotopic ions until 3 Da
Alignment	RT tolerance 0.05 min<br>MS1 tolerance 0.01 Da<br>RT factor 0.5<br>MS1 factor 0.5<br>gap filling

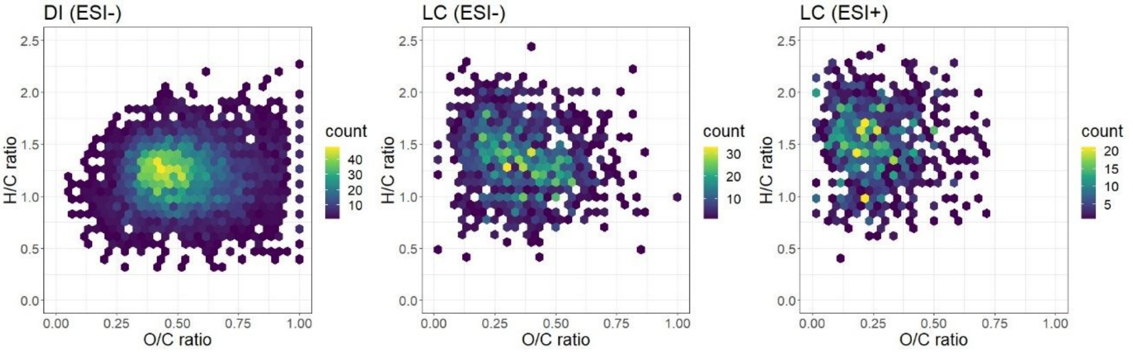

Figure S1. Van Krevelen plots show the distribution of the molecular formulas assigned in DI, LC (ESI-), and LC (ESI+). Each hexagon represents one or more molecular formulas, with color serving as a quantitative measure of the number of formulas overlapping in different regions of the plot.

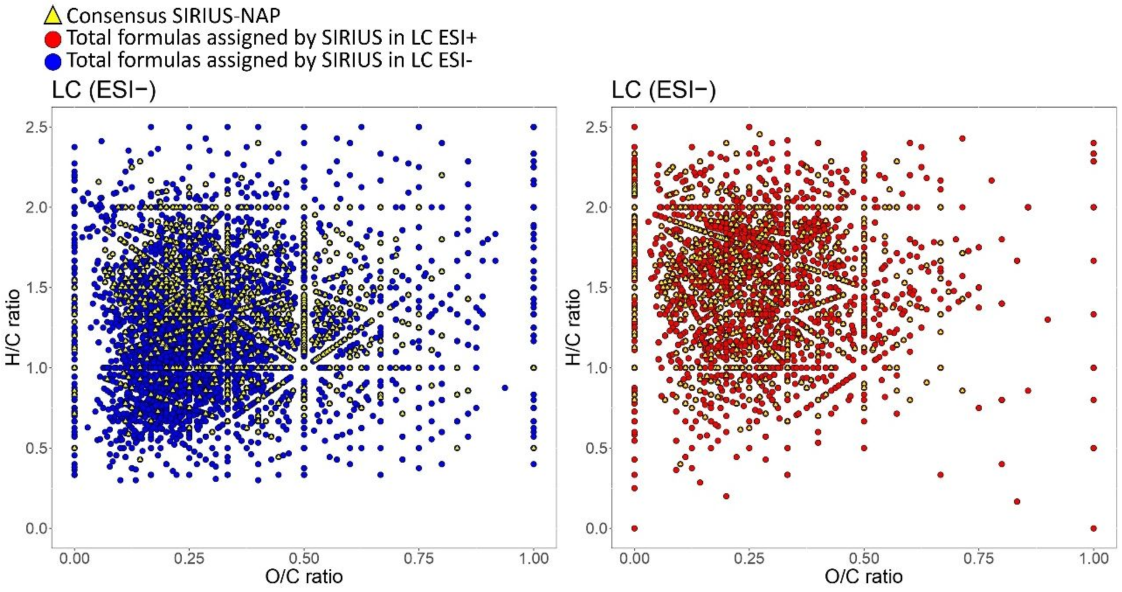

Figure S2. Van Krevelen plots show the total number of molecular formulas assigned by SIRIUS in LC-HRMS ESI negative (blue) and ESI positive (red) ion mode. In yellow, formulas that found consensus in NAP and so have also structure prediction.

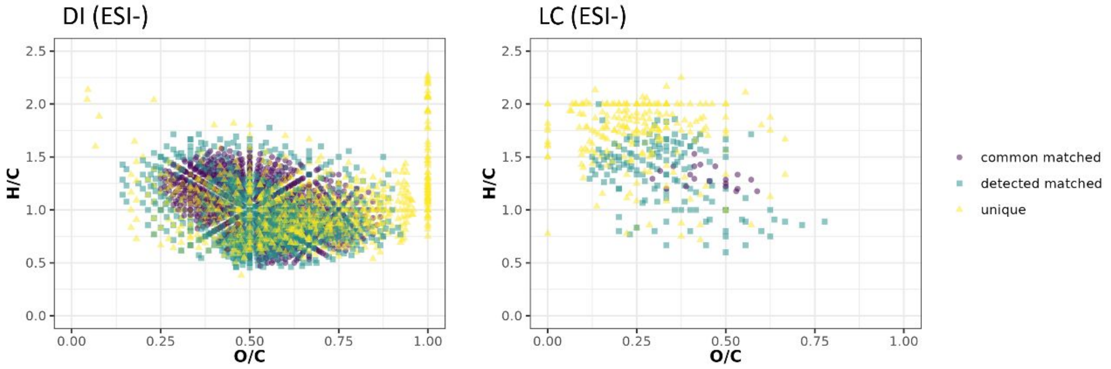

Figure S3. Van Krevelen plot shows the results of SRFA analysis by DI-HRMS and LC-HRMS workflow in comparison with the InterLabStudy. Green squares represent the matches between our results and ions that were detected and assigned by at least three instruments tested in the InterlabStudy (22% in DI and 4% in LC). Purple circles represent the matches between our results and assigned ions common to all instruments tested in the InterlabStudy (95% in DI and 2% in LC). Yellow triangles represent the ions that uniquely figure in our results. These plots were directly downloaded from the InterLabStudy comparison platform (https://kairos.warwick.ac.uk/InterLabStudy).

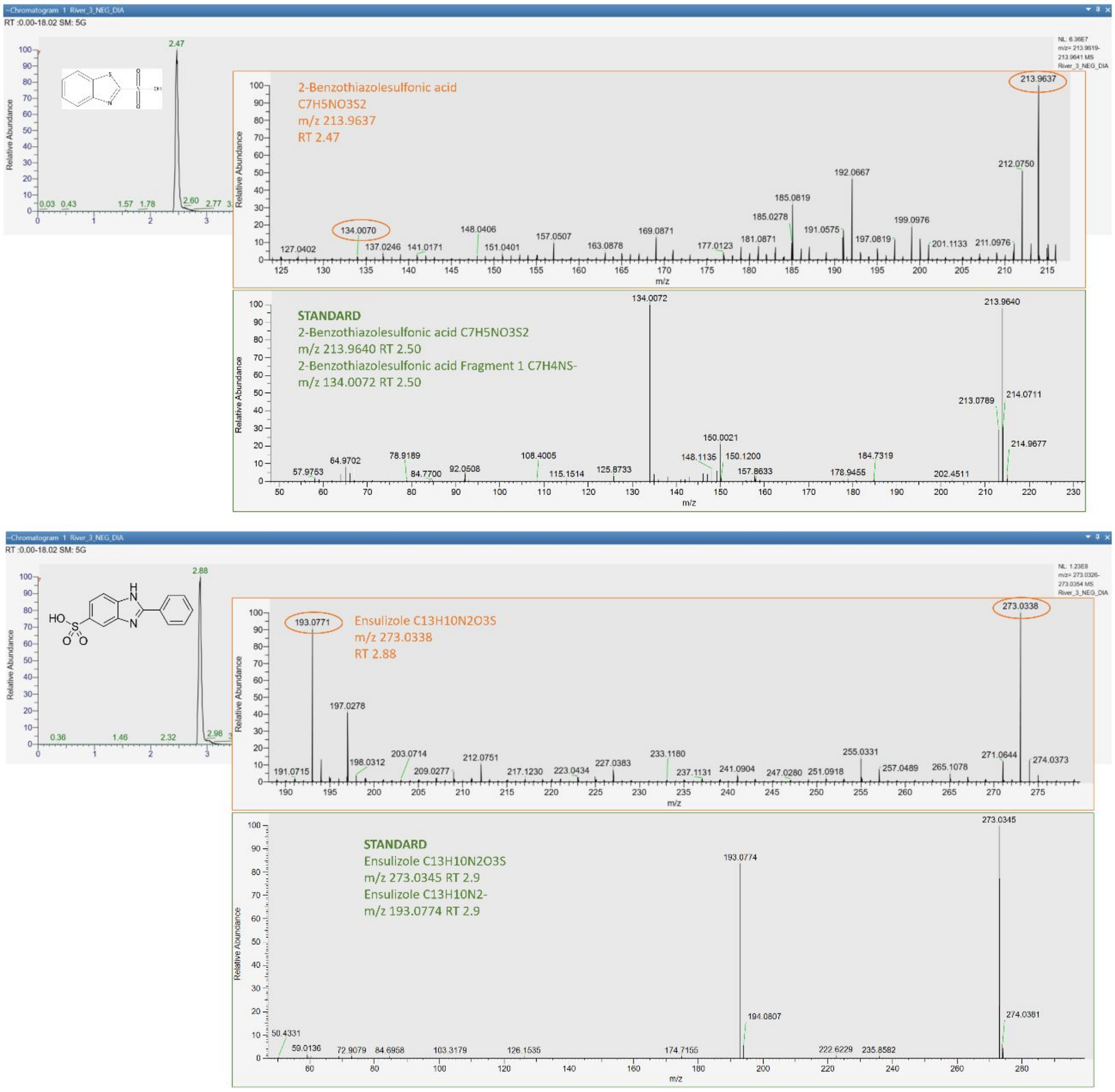

Figure S4. Confirmed identification of Ensulizonle and 2-Benzothiazole sulfonic acid. Comparisons of LC-HRMS molecular features detected in river samples to authentic standards analyzed under the same conditions and acquired in DDA mode. The identifications were confirmed (Level 1) by comparing the major fragment ions patterns (sample in orange and standard in green).
